# Effects of cardiorespiratory fitness and body mass index on cardiometabolic risk factors in schoolchildren

**DOI:** 10.1186/s12887-023-04266-w

**Published:** 2023-09-09

**Authors:** Natália Carvalho Bagatini, Carolina Dertzbocher Feil Pinho, Gabriela Tomedi Leites, Rogério da Cunha Voser, Anelise Reis Gaya, Giovani dos Santos Cunha

**Affiliations:** 1 School of Physical Education, Physiotherapy and Dance, Exercise Research Laboratory, Universidade Federal do Rio Grande do Sul, Porto Alegre, Brazil; 2 Federal University of Health Sciences of Porto Alegre, Porto Alegre, Brazil

**Keywords:** Children, Fitness, Cardiometabolic health, Blood pressure

## Abstract

**Introduction:**

High levels of physical fitness established during childhood and adolescence have been associated with positive effects on cardiometabolic risk factors (CMRF), which persist into adulthood. Conversely, a sedentary lifestyle, overweight, and obesity during this period are considered public health problems. These conditions tend to worsen in adulthood, increasing the incidence of chronic diseases, deteriorating CMRF, and consequently leading to higher comorbidity and mortality rates.

**Objective:**

To investigate the effect of cardiorespiratory fitness (CRF) and body mass index (BMI) on CMRF in children and adolescents.

**Methods:**

The sample consisted of 49 schoolchildren of both sexes aged 10–17 years. Anthropometric assessments, CRF test, muscle strength test, and blood pressure (BP) measurement were conducted. Participants were allocated into groups based on BMI (eutrophic, overweight, obese), and CRF levels (low-fit, normal-fit, and high-fit).

**Results:**

Obese individuals had lower CRF values compared to the eutrophic and overweight groups. The cardiometabolic risk profile (CMRP) was significantly higher in the obese group compared to the eutrophic group but showed no significant difference compared to the overweight group. The hight-fit group had lower CMRP values compared to the low-fit group.

**Conclusions:**

Higher BMI and CRF values had negative and positive effects on CMRF and CMRP in schoolchildren, respectively. Overweight or obese schoolchildren with low levels of CRF constitute an unfavourable cardiometabolic risk profile.

## Introduction

High levels of fitness and physical activity in childhood and adolescence can positively contribute to the development of a healthier lifestyle in adulthood and reduce the risk of developing chronic diseases [1, 2]. Moreover, childhood is a critical phase for learning, and numerous studies have demonstrated that exercise and physical activity during this period positively impact cognitive, motor, and communicative development [1, 3–6]. Regular exercise and physical activity during early life offer several benefits, including the protection of bone, cardiometabolic, and mental health. They also reduce the risk of developing chronic diseases and comorbidities in adulthood, such as hypertension, diabetes, obesity, and metabolic syndrome, ultimately leading to reduced mortality [5, 7–18]. Similarly, high levels of physical fitness (muscular and cardiorespiratory fitness, body composition, strength, speed, and muscle power) have been positively associated with a better quality of life, improved academic performance and psychological profile (depression, anxiety, mood state). Conversely, high levels of physical fitness have been negatively associated with cardiometabolic risk factors (CMRF) in children and adolescents [19–22].

In contrast, a sedentary lifestyle and physical inactivity have been identified as the primary causes of various diseases that impact millions of individuals. The development of these diseases typically takes place over an extended period, with some starting as early as preschool age (3–5 years) or even during pregnancy [23]. Consequently, individuals who are sedentary and overweight during childhood have a higher likelihood of experiencing cardiovascular diseases, obesity, diabetes, depression, and breast cancer during adulthood [23–26]. Recent years have witnessed an increase in sedentary behaviour and reduced physical activity levels among children and adolescents. This trend can be primarily attributed to the decline in public spaces available for physical activities, rising urban violence, cost of living, increased access to technology (television, video games, computer), excessive screen time, and consumption of high-fat foods (fast food) [27, 28]. Furthermore, these factors have negative implications for adult health [23–25] and are now recognized as a significant public health concern. However, it is known that over 90% of school-aged children and adolescents fail to meet international recommendations for daily physical activity. This low level of physical activity significantly contributes to the prevalence obesity, affecting schoolchildren as well [4, 29]. According to the World Health Organization, in 2020, there were over 39 million overweight or obese children under the age of five globally [30]. These statistics clearly indicate an epidemic of childhood obesity, highlighting the urgent need to identify its causes and potential solutions in order to address this significant global health issue.

In Brazil, the prevalence of obesity among children under the age of five is currently 5%, and this rate is increasing by 0.5% annually. For children and adolescents aged 5–17 years, the prevalence of overweight and obesity can range between 35% and 40% [23]. Startling, there has been a 300% rise in overweight and obesity rates over the past decade [7] and approximately 50% of Brazilian children fail to meet the minimum recommendations for physical activity [5].

For all the aforementioned reasons, this study aimed to investigate the effect of cardiorespiratory fitness (CRF) and body mass index (BMI levels) on health indicators in children and adolescents.

## Methods

### Sample

Forty-nine children and adolescents, of both sexes, aged between 10 and 17 years were included in the study. The individuals were allocated into groups based on their sex, BMI, and physical fitness. Group allocation based on BMI followed the CDC Growth Charts using the following percentile cut-points: <84th percentile for eutrophic, 85th to 94th percentile for overweight, and ≥ 95th percentile for obese children and adolescents [31, 32]. Group allocation based on CRF was conducted following the approach outlined by Tomkinson et al. 2017 using percentile cut-points as follows: <30th percentile for low-FIT, 30th to 80th percentile for normal-FIT, and > 80th percentile for high-FIT [33].

The study was approved by the Research Ethics Committee of the Federal University of Rio Grande do Sul (CAAE: 40235214.4.00005347) prior to participating in the study, all participants and their legal representatives signed the Informed Consent Form and the Informed Term of Consent. The study was conducted in accordance with the Declaration of Helsinki.

### Experimental procedures

Data collection occurred during a single visit to the participants’ school in the following order: [1] measurement of systolic blood pressure (SBP), diastolic blood pressure (DBP), and mean arterial pressure (MAP) measured twice with the subjects sitting and at rest for at least 10 min by an automatic sphygmomanometer (electronic automatic-measurement arterial blood pressure device - Omron-HEM-7122) placed on the left arm; [2] anthropometric assessment of height, sitting height (WISO stadiometer, 1 mm resolution), body mass (G-TECH scale, model Glass 3 control, 0.05 kg; G Tech Technology Ltd), waist circumference (WC), hip circumference (HC) (Sanny measuring tape, São Paulo, Brazil), waist-to-stature ratio (WC/S), waist-to-hip ratio (WC/H), peak height velocity (PHV) [34], BMI, fat percentage (%F), and fat-free mass [35]. Body composition was assessed by skinfolds measurement (Mitutoyo-CESCORF plicometer, Porto Alegre-RS) according to the equation proposed by Slaughter et al. [36]. The anthropometric assessment followed the ISAK recommendations; [37] muscular fitness evaluation was performed through the handgrip test using a hand dynamometer (handgrip - Sanny). The dynamometer was adjusted according to the size of the student’s hand, who performed two maximum handgrip repetitions lasting 5 s with the dominant hand, with a 30-second interval between repetitions. The highest value reached was considered the maximum handgrip strength; [4] CRF assessment was determined from the 20-meters Shuttle Run test developed by Leger et al. [38], together with the use of a frequency meter (Team Polar, Polar Electro Oy, Kempele, Finland) to measure the maximum heart rate (HR_max_). The total distance covered during the test was converted into VO_2peak_ values (ml.kg^− 1^.min^− 1^). The cardiometabolic risk profile (CMRP) was calculated from the following equation: CMRP = (VO_2peak_ + Handgrip) × (-1) + (BMI + MAP + %FM + WC + HC + WC/S + WC/H).

### Statistical analysis

Data expressed as mean and standard deviation. Normality of the data distribution was evaluated using the Shapiro-Wilk test, and homoscedasticity of the variables was assessed using Levene’s test. Differences among groups allocated by BMI levels and CRF were established by analysis of variance (ANOVA) with Bonferroni post hoc. Effect size (ES) was determined by partial eta squared (η^2^), with values of 0.01, 0.06, and above 0.15 considered small, medium, and large effects, respectively [13]. Comparisons between sexes were performed using Independent T-Test and its ES was determined by Cohen d and interpreted as follows: < 0.20 (trivial), 0.2–0.59 (small), 0.60–1.19 (moderate), 1.20–1.99 [39], 2.0–3.9 (very large), > 4.0 (near perfect) [40]. The significance level adopted was p < 0.05.

## Results

Table 1 presents the physical and cardiometabolic characteristics of the children and adolescents. The data reveals that boys exhibited significantly higher values of VO_2peak_ and total distance covered during the Shuttle Run Test in comparison to girls. In addition, boys demonstrated higher FFM and WC/H Ratio compared to girls.

The analysis of physical and cardiometabolic variables based on BMI groups is presented in Table 2. As expected, overweight and obese individuals displayed higher body mass (kg) and BMI compared to their eutrophic peers. Moreover, obese individuals exhibited lower values of total distance covered in the Shuttle Run Test (p = 0.002) and VO_2peak_ (p = 0.020) compared to eutrophic individuals, respectively. Fat mass (% and kg) and BFI were significantly higher in the overweight and obese groups compared to the eutrophic group. Furthermore, WC was significantly higher in the obese group, while HC was significantly higher in the overweight group, compared to the other groups. The WC/S Ratio was significantly higher in the obese group compared to the eutrophic group (p = 0.002). Similarly, the WC/H Ratio demonstrated significantly higher values favouring the obese group over the overweight and eutrophic groups (p = 0.001 and p = 0.019, respectively). Additionally, the obese group exhibited higher CMRP values compared to the eutrophic group (p = 0.002).

When children and adolescents were allocated into groups based on their CRF levels (Table 3), the High-Fit group demonstrated significantly lower CMRP values compared to the Low-Fit group (p = 0.038).

**Table 1:** Physical, anthropometrical and cardiometabolic characteristics of children and adolescents

Variable	Girls (n = 15)	Boys (n = 33)	P-value	ES	ESq
Age (years)	13.0 ± 2.0	12.7 ± 2.2	0.666	0.142	Trivial
Body mass (kg)	47.5 ± 13.3	48.9 ± 14.5	0.755	0.100	Trivial
Height (m)	1.49 ± 0.11	1.55 ± 0.13	0.127	0.498	Small
BMI (kg/m^2^)	21.0 ± 3.9	19.8 ± 3.6	0.311	0.319	Small
PHV (years)	0.48 ± 1.70	-1.03 ± 1.84*	0.010	0.826	Moderate
Handgrip (kgf)	23.4 ± 8.2	27.4 ± 12.5	0.279	0.378	Small
Handgrip (kgf.FFM^− 1^)	0.67 ± 0.16	0.70 ± 0.20	0.720	0.165	Trivial
VO_2peak_ (m.kg^− 1^.min^− 1^)	40.3 ± 5.6	45.7 ± 5.1*	0.003	1.008	Moderate
Shuttle Run (m)	462 ± 270	763 ± 443*	0.024	0.820	Moderate
SBP (mmHg)	110 ± 13.2	112 ± 13.5	0.533	0.149	Trivial
DPB (mmHg)	64 ± 6.3	61 ± 6.7	0.096	0.461	Small
MAP (mmHg)	80 ± 7.8	78 ± 7.1	0.517	0.268	Small
Fat mass (%)	27.2 ± 10.1	21.0 ± 11.0	0.069	0.584	Small
Fat mass (kg)	14.0 ± 8.7	10.6 ± 8.3	0.199	0.399	Small
FFM (kg)	33.4 ± 5.8	38.7 ± 11.1*	0.089	0.598	Small
BFI (kg.m^− 2^)	6.0 ± 3.3	4.3 ± 3.1	0.093	0.530	Small
BFFMI (kg.m^− 2^)	14.9 ± 1.2	15.1 ± 3.8	0.885	0.070	Trivial
WC (cm)	62.2 ± 7.2	65.4 ± 9.0	0.298	0.392	Small
HC (cm)	84.7 ± 11.2	83.1 ± 9.0	0.616	0.157	Trivial
Ratio WC/S (cm)	0.42 ± 0.04	0.42 ± 0.05	0.984	0.000	Trivial
Ratio WC/H (cm)	0.74 ± 0.05	0.78 ± 0.07*	0.041	0.657	Moderate
CMRP	1.72 ± 4.8	-1.15 ± 7.6	0.190	0.451	Small

Data expressed as mean and standard deviation (mean ± SD), where BMI = body mass index; PHV = peak height velocity; SBP = systolic blood pressure; DPB = diastolic blood pressure; MAP = mean arterial pressure; FFM = fat free mass; BFI = body fat index; BFFMI = Body fat free mass index; WC = waist circumference; S = Stature; HC = hip circumference; H = hip; CMRP = cardiometabolic risk profile; ES = Cohen d effect size; ES_q_ = qualitative effect size. * = Significantly different from girls’ group. Significance (P < 0.05)

**Table 2:** Physical, anthropometrical and cardiometabolic characteristics of children and adolescents according to body mass index

Variable	Eutrophic (n = 35)	Overweight (n = 7)	Obese (n = 6)	P-Value	ES	ES_q_
Age (years)	12.9 ± 2.1	13.2 ± 2.7	11.5 ± 2.0	0.285	0.054	Small
Body mass (kg)	44.4 ± 11.4	57.6 ± 14.8*	61.4 ± 14.0*	0.003	0.233	Large
Height (m)	1.53 ± 0.14	1.53 ± 0.13	1.52 ± 0.08	0.993	0.000	Small
BMI (kg.m^− 2^)	18.5 ± 2.0	23.9 ± 2.3*	25.9 ± 4.3*	< 0.001	0.593	Large
PHV (years)	-0.62 ± 1.84	0.1 ± 2.34	-1 ± 2.03	0.868	0.025	Small
Handgrip (kgf)	26.7 ± 11.8	28.6 ± 10.3	21.0 ± 9.8	0.061	0.034	Small
Handgrip (kgf.FFM^− 1^)	0.72 ± 0.18	0.66 ± 0.10	0.53 ± 0.19	0.463	0.122	Medium
VO_2peak_ (ml.kg^− 1^.min^− 1^)	45.6 ± 5.7	41.1 ± 4.4	39.6 ± 4.2*	0.020	0.170	Large
Shuttle Run (m)	795 ± 410	488 ± 262	203 ± 89*	0.002	0.260	Large
SBP (mmHg)	112 ± 14	114 ± 13	109 ± 9	0.837	0.008	Small
DPB (mmHg)	62 ± 6.6	61 ± 4.6	64 ± 9.8	0.834	0.008	Small
MAP (mmHg)	78 ± 7.0	79 ± 6.6	78 ± 10.2	0.992	0.000	Small
Fat mass (%)	19.4 ± 8.0	29.8 ± 8.0*	34.8 ± 17.2*	< 0.001	0.286	Large
Fat mass (kg)	8.5 ± 3.2	17.6 ± 6.8*	22.9 ± 16.2*	< 0.001	0.410	Large
FFM (kg)	36.2 ± 10.5	39.9 ± 9.7	34.4 ± 7.6	0.641	0.020	Medium
BFI (kg.m^− 2^)	3.6 ± 1.4	7.2 ± 2.2*	9.5 ± 5.9*	< 0.001	0.447	Large
BFFMI (kg.m^− 2^)	14.5 ± 3.4	16.6 ± 1.6	16.3 ± 2.7	0.157	0.079	Medium
WC (cm)	62.5 ± 4.9	64.2 ± 12.8	74.1 ± 12.9*	0.005	0.211	Large
HC (cm)	81.3 ± 7.0	91.6 ± 11.0*	87.7 ± 15.6	0.017	0.168	Large
WC/S Ratio (cm)	0.40 ± 0.03	0.42 ± 0.06	0.48 ± 0.08*	0.002	0.240	Large
WC/H Ratio (cm)	0.77 ± 0.04	0.71 ± 0.09	0.84 ± 0.08*#	0.001	0.258	Large
CMRP	-2.20 ± 5.7	2.57 ± 6.3	7.78 ± 8.4*	0.002	0.251	Large

Data expressed as mean and standard deviation (mean ± SD), where BMI = body mass index; PHV = peak height velocity; SBP = systolic blood pressure; DPB = diastolic blood pressure; MAP = mean arterial pressure; FFM = fat free mass; BFI = body fat index; BFFMI = Body fat free mass index; WC = waist circumference; S = Stature; HC = hip circumference; H = hip; CMRP = cardiometabolic risk profile; ES = partial eta square effect size; ES_q_ = qualitative effect size; P-value = Corresponding value for the intergroup comparison using one-way ANOVA. *Significantly different from eutrophic group. # Significantly different from overweight group. Significance (P < 0.05)

**Table 3:** Physical and cardiometabolic characteristics of children and adolescents according to cardiorespiratory fitness

Variable	Low-Fit (n = 13)	Normal-Fit (n = 21)	High-Fit (n = 11)	P-Value	ES	ES_q_
Age (years)	11.4 ± 2.2	13.7 ± 1.9*	12.7 ± 1.9	0.010	0.196	Large
Body mass (kg)	50.1 ± 16.6	50.2 ± 12.5	44.1 ± 12.4	0.457	0.037	Small
Height (m)	1.48 ± 0.10	1.57 ± 0.11	1.51 ± 0.15	0.118	0.097	Medium
BMI (kg.m^− 2^)	22.2 ± 4.8	20.0 ± 3.4	18.7 ± 2.2	0.073	0.117	Medium
PHV (years)	-1.37 ± 1.98	0.11 ± 1.78	-0.80 ± 1.89	0.202	0.114	Medium
Handgrip (kgf)	22.4 ± 11.6	29.3 ± 11.1	25.3 ± 12.3	0.252	0.065	Medium
Handgrip (kgf.FFM^− 1^)	0.62 ± 0.21	0.73 ± 0.16	0.71 ± 0.21	0.274	0.061	Medium
VO_2peak_ (ml.kg^− 1^.min^− 1^)	40.6 ± 3.8	42.3 ± 4.4*	51.5 ± 2.8*#	< 0.001	0.561	Large
Shuttle Run (m)	258 ± 105	637 ± 239*	1154 ± 346*#	< 0.001	0.659	Large
SBP (mmHg)	109 ± 11	114 ± 14	114 ± 13	0.435	0.039	Small
DPB (mmHg)	63 ± 8	61 ± 6	62 ± 5	0.701	0.017	Small
MAP (mmHg)	78 ± 8	79 ± 6	80 ± 7	0.949	0.002	Small
Fat mass (%)	27.5 ± 15.0	21.4 ± 7.2	21.0 ± 12.0	0.254	0.063	Medium
Fat mass (kg)	15.0 ± 13.3	11.3 ± 6.0	8.7 ± 4.0	0.192	0.076	Medium
FFM (kg)	35.0 ± 9.5	38.9 ± 8.2	35.3 ± 12.5	0.449	0.037	Small
BFI (kg.m^− 2^)	6.5 ± 5.0	4.4 ± 2.2	3.8 ± 2.0	0.105	0.102	Medium
BFFMI (kg.m^− 2^)	15.6 ± 2.7	15.5 ± 1.7	14.8 ± 3.1	0.688	0.018	Small
WC (cm)	66.9 ± 13.0	64.0 ± 6.2	63.2 ± 5.6	0.530	0.030	Small
HC (cm)	83.7 ± 11.3	84.7 ± 9.3	81.5 ± 8.7	0.734	0.015	Small
WC/S Ratio (cm)	0.45 ± 0.07	0.40 ± 0.03	0.41 ± 0.03	0.071	0.118	Medium
WC/H Ratio (cm)	0.79 ± 0.10	0.75 ± 0.05	0.77 ± 0.04	0.314	0.054	Small
CMRP	3.24 ± 7.9	-0.51 ± 3.5	-1.98 ± 2.9*	0.038	0.144	Medium

Data expressed as mean and standard deviation (mean ± SD), where BMI = body mass index; PHV = peak height velocity; SBP = systolic blood pressure; DPB = diastolic blood pressure; MAP = mean arterial pressure; FFM = fat free mass; BFI = body fat index; BFFMI = Body fat free mass index; WC = waist circumference; S = Stature; HC = hip circumference; H = hip; CMRP = cardiometabolic risk profile; ES = partial eta square effect size; ES_q_ = qualitative effect size; P-value = Corresponding value for the intergroup comparison using one-way ANOVA. Significantly different from Low-Fit group. # Significantly different from Normal-Fit group. Significance (P < 0.05)

## Discussion

The main findings of the study were: [1] there is an effect of sex on CRF in favor of boys; [2] BMI has an effect on CMRF, with the obese group presenting higher CMRP values in comparison to the eutrophic group; [3] high levels of CRF have a protective effect on CMRP, as the High-fit group demonstrated lower values compared to the Low-Fit group; [4] the obese group had similar CRF values compared to the overweight group; [5] no significant effects of sex, BMI and CRF levels on muscular fitness (handgrip) were identified.

Recently, it has been suggested that the higher levels of cardiorespiratory and muscular fitness observed in boys compared to girls can be attributed to the fact that boys have higher levels of physical activity and FFM [41]. The present study partially supports this evidence, as no significant differences were found for muscular fitness, despite the higher FFM values observed in boys compared to girls. The variations in physical fitness between the sexes could be partially explained by genetic factors, environmental influences, individual differences, and differences in biological maturation levels [42].

BMI is a widely used health indicator in the pediatric population. Upon grouping the schoolchildren based on their BMI, it was observed that the obese group had lower CRF, as measured by the total distance covered in the Shuttle Run Test and VO_2peak_, compared to the eutrophic group. However, the CRF of the obese group was similar to that of the overweight group. The Shuttle Run Test (a 20-meter running test) is the most commonly used protocol for the assessing CRF in children and youth [43]. According to a systematic review conducted by Lang et al. (2018), the Shuttle Run Test was found to be associated with various health indicators, with higher values of distances covered being linked to lower levels of adiposity [44]. This finding is consistent with the present study, as individuals with higher percentages of body fat exhibited lower performance in the Shuttle Run Test. A study conducted by Artero et al. (2010) obtained similar results using the Shuttle Run and handgrip tests [41]. In the run test, obese boys achieved significantly lower distances compared to both the overweight and eutrophic groups. Additionally, the overweight group also achieved lower distances compared to the eutrophic group. Lower results were observed in obese girls compared to the eutrophic group, while no statistical differences were found in the overweight group. The findings from the present study align with those of the Shuttle Run test. However, it is important to note that for BMI results, boys and girls were grouped together in the same categories of eutrophic, overweight, and obese groups. In the analysis of the handgrip test, both obese boys and girls achieved higher values compared to the eutrophic and overweight groups. Furthermore, the overweight group also obtained higher results compared to the eutrophic group. The authors of the aforementioned study suggest that these higher results in the overweight and obese groups can be attributed to their higher FFM values. These elevated FFM values may be a response to the excess body fat, as the body increases fat-free mass to support the body weight. In the present study, the lack of differences between the groups in the handgrip test could potentially be attributed to the absence of differences for FFM. Casonatto et al. (2015) similarly observed poorer performances in obese and overweight boys and girls during the CRF test (9-minute walk/run test) [45]. This suggests that there is an association between poor performance and health indicators among these populations [46].

In addition to the findings regarding physical fitness, the study observed higher CMRP values in the obese group compared to the eutrophic group, with no significant differences from the overweight group. Existing literature reports that overweight/obese individuals tend to exhibit deteriorated cardiometabolic health indicators, indicated by higher CMRP values compared to their eutrophic peers. As obesity represents a significant cardiometabolic risk factor associated with increased mortality, it is crucial to examine whether children and adolescents already display this unfavourable CMRP, which can lead to the development of several comorbidities in adulthood, thereby compromising the long-term health of these individuals [47, 48]. This study established that both obese and overweight individuals have higher CMRP values compared to eutrophic, further confirming the negative effects of high adiposity levels on CMRP and the cardiometabolic health of schoolchildren.

When the individuals were allocated based on their CRF levels (low-fit, normal-fit, and high-fit), it was observed that the High-Fit group had better CMRP values compared to the Low-Fit group, while the values were similar to those of the Normal-Fit group. This finding indicates that low CRF has an adverse effect on the cardiometabolic health of schoolchildren. However, higher BMI and CRF values had contrasting effects on CMRF and CMRP in schoolchildren. Higher BMI values were associated with negative effects on CMRF and CMRP, while higher CRF values were associated with positive effects on CMRP. In addition, overweight or obese schoolchildren with low levels of CRF constitute an unfavourable CMRP. Furthermore, it is important to note that CMRP was calculated through the integration of various CMRFs. This approach enables the identification of cardiometabolic risks to health caused by obesity and low levels of CRF, which often cannot be established through separate analyses of health indicators.

The present study has both strengths and limitations. Among the strengths are the utilization of a test battery for assessing CMRF and CMRP that is simple to perform, has low financial cost, and can be applied to large populations. This battery effectively identified health risks associated with obesity and highlighted the protective effects of high CRF levels. Nevertheless, the study does have limitations, most notably a relatively small sample size, especially within the overweight and obese groups. This could potentially introduce bias and impede the precise identification of the effects of CRF and BMI on CMRF among schoolchildren. Consequently, the results of this study should be interpreted with caution.

For practical application, it is recommended to assess schoolchildren longitudinally throughout their school years using a comprehensive test battery capable of detecting CMRF and consequently CMRP. This approach can help mitigate the onset and severity of chronic diseases at an early stage. Furthermore, aiming to enhance physical fitness levels and decrease adiposity should be a primary objective to enhance the cardiometabolic health of schoolchildren.

## Conclusion

Obesity and CRF had negative and positive effects on CMRF and CMRP in schoolchildren, respectively. Overweight or obese schoolchildren with low levels of CRF exhibit an unfavourable CMRP. High levels of CRF seems to exert a protective effect on cardiometabolic health of schoolchildren.

## Data Availability

The database used and analyzed in the present study is not publicly available as its information may compromise the participants’ privacy and consent involved in the research. However, the data are available from the corresponding author, upon request.

## List of abbreviations

%F: Fat percentage
BFFMI: Body fat free mass index
BFI: Body fat index
BMI: Body mass index
BP: Blood pressure
CMRF: Cardiometabolic risk factors
CMRP: Cardiometabolic risk profile
CRF: Cardiorespiratory fitness
DBP: Diastolic blood pressure
ES: Effect size
Esq: Qualitative effect size
FFM: Fat-free mass
HC: Hip circumference
HR_MAX_: Maximum heart rate
MAP: Mean arterial pressure
PHV: Peak height velocity
SBP: Systolic blood pressure
VO_2peak_: Peak oxygen consumption
WC: Waist circumference
WC/H Ratio: Waist-to-hip ratio
WC/S Ratio: Waist-to-stature ratio
